# Nicolau syndrome due to diclofenac sodium (Voltaren®) injection: a case report

**DOI:** 10.1186/1752-1947-8-404

**Published:** 2014-12-04

**Authors:** İnci Kılıç, Füruzan Kaya, Ayşe T Özdemir, Tuğba Demirel, İlhami Çelik

**Affiliations:** Department of Infectious Disease, Education and Research Hospital, Sanayi District. Atatürk Boulevard. Hospital Street. No: 78, 38010 Kocasinan, Kayseri, Turkey

**Keywords:** Diclofenac sodium, Intragluteal injection, Nicolau syndrome

## Abstract

**Introduction:**

Nicolau syndrome, also known as livedo-like dermatitis or embolia cutis medicamentosa, is a rare complication following the intramuscular or intra-articular injection of various drugs.

**Case presentation:**

In our case report we report the case of a 45-year-old Turkish woman who developed Nicolau syndrome after an intramuscular injection in her right gluteal region of single-dose diclofenac sodium to treat a headache. A culture taken from the ulcer showed growth of methicillin-sensitive *Staphylococcus aureus* on the 10^th^ day. The secondary staphylococcal infection was treated effectively with intravascular ampicillin-sulbactam (4×1.5g/day). She was treated with surgical debridement, sterile dressings and analgesics. The ulcer healed completely within 12 weeks with scarring.

**Conclusions:**

Although Nicolau syndrome develops very rarely, it is an important cause for morbidity. It is an iatrogenic condition, treated mostly by health care workers. Thus, although it appears to be a very simple procedure for a health care worker, care must be taken during intramuscular injections. Although diclofenac sodium is a widely used non-steroidal anti-inflammatory drug, Nicolau syndrome following intramuscular diclofenac sodium injection has rarely been reported in the published literature. The application of a cold compress was considered to be an aggravating factor in our patient. This case highlights the need for awareness about this condition and the need to exercise utmost care during the administration of any parenteral injections by health workers.

## Introduction

Embolia cutis medicamentosa, or Nicolau syndrome (NS), was first described in 1924 after an intragluteal injection of bismuth salts for the treatment of syphilis [1]. According to one theory, NS occurs when an intramuscular medication is inadvertently injected directly in an arterial lumen or wall, leading to vessel thrombosis and subcutaneous tissue and muscle necrosis [2]. The signs of skin discoloration are usually accompanied by severe pain and extensive inflammation [3]. Typically, necrosis develops following hyperemia, skin discoloration, livedoid dermatitis and hemorrhagic patch formation at the injection site [3]. Severe cases may take a rapid clinical course and lead to death [4].

We report a case of NS following an intramuscular injection of diclofenac sodium (Voltaren®).

## Case presentation

A 45-year-old Turkish woman presented to our clinic with skin discoloration in her right gluteal region, and a physical examination revealed a skin necrosis with a size of approximately 15×20cm. She had received an intramuscular (intragluteal) injection of diclofenac sodium (Voltaren®) for treatment of a headache prior to the onset of the skin necrosis. After receiving diclofenac sodium, she immediately experienced severe pain followed by blistering and ulceration. On the second day post-injection, her skin turned dark purple with a hemorrhagic patch. By the 10th day post-injection, the erythematic area had decreased, but most of the darkly colored skin had progressively turned black. Three weeks later, the black skin had changed into an eschar. There was no other drug intake or systemic illness.

**Figure 1:**
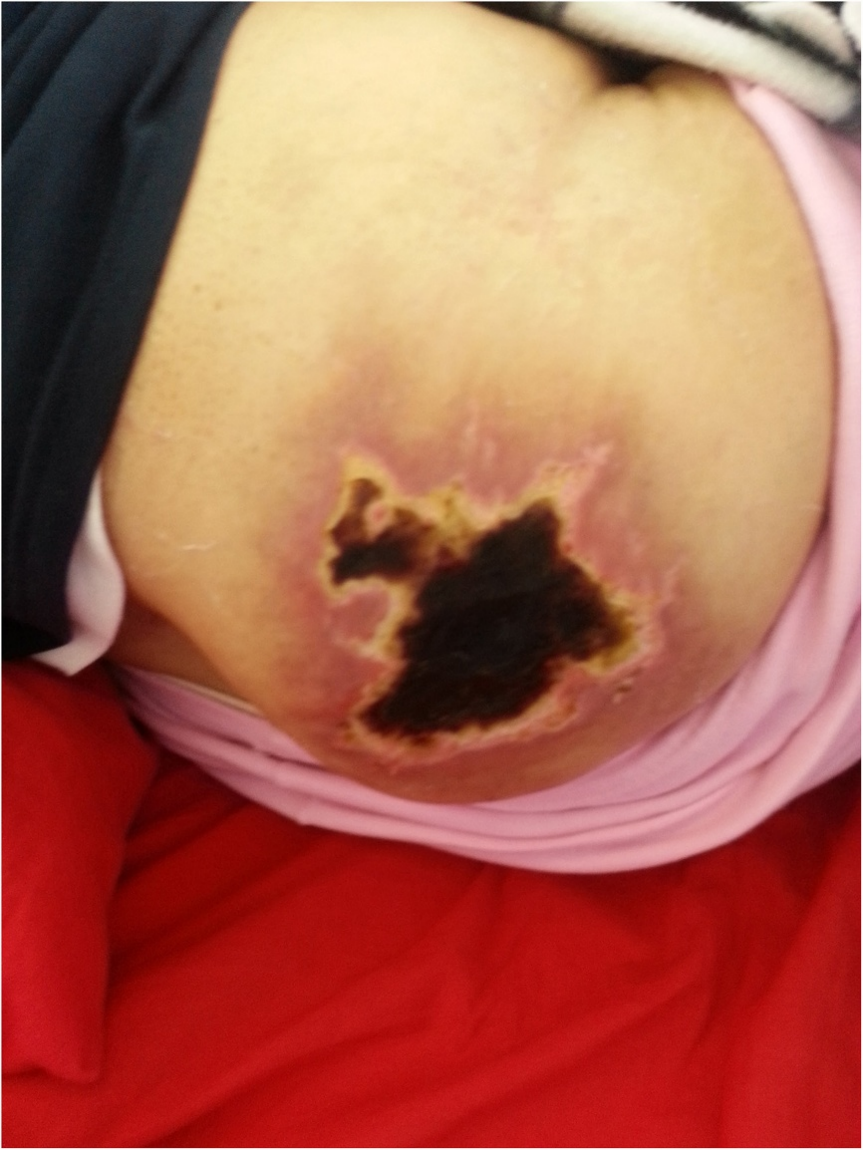
**Photograph of right gluteal lesion 3-weeks post-injection illustrating the nature and extent of the eschar.**

A cutaneous examination showed a large, tender, non-indurated ulcer with necrotic eschar covering almost the entire right gluteal region with minimal extension to the left (Figure 1). Other cutaneous and systemic examinations were normal. There was no regional or generalized lymphadenopathy.

A complete blood count, including bleeding and clotting time, and urine examinations were normal. Her chest X-ray, blood urea, serum creatinine, liver function tests and creatine kinase, were normal. The results of her Venereal Disease Research Laboratory (VDRL) and human immunodeficiency virus (HIV)-1 and HIV-2 tests were negative. A superficial ultrasonography showed diffuse edema, with sparing of the muscle and no fluid collections. The fluid found and specimens of infected deep soft tissues were sent for immediate Gram’s stain, culture and antibiotic sensitivity tests. The Gram’s stain revealed numerous polymorphonuclear cells. A culture from the ulcer showed growth of methicillin-sensitive *Staphylococcus aureus*. The secondary staphylococcal infection was treated effectively with intravascular ampicillin-sulbactam (4×1.5g/day) for 21 days. She was treated with surgical debridement, sterile dressings, and analgesics. The ulcer healed completely within 12 weeks with scarring.

## Discussion

Firstly described by Freudenthal and Nicolau in 1924, typically NS presents with pallor, owing to a local reflex vasospasm, and pain, rapidly followed by erythema, hemorrhagic patch, blistering, and variable degree of necrosis [5]. NS has been reported with the administration of various other drugs such as penicillins, local anesthetics, corticosteroids and non-steroidal anti-inflammatory drugs (NSAIDs) in the literature [6].

According to Saputo and Bruni [7] the syndrome is more frequent in the pediatric population, mainly in children who are younger than 3 years, in which the phenomenon of artery embolism may be more likely to happen due to the smaller size of the vascular segments involved [7].

The pathogenesis of the disease is not known, but there have been several hypotheses. First, it is presumed that the sympathetic nerve is stimulated by pain from the intra-arterial or periarterial injection of drugs, causing vasospasms and leading to ischemia. Second, this is related to the pharmacologic properties of NSAIDs. NSAIDs inhibit prostaglandin synthesis by the inhibition of cyclooxygenase. Ischemic necrosis occurs after the vasospasms are induced by the drug’s suppression of prostaglandin. Third, the intra-arterially injected drug causes embolic occlusion. Nicolau histologically discovered bismuth salts in the peripheral arteries of his patient. Fourth, ischemic necrosis progresses from vascular rupture due to perivascular inflammation from a cytotoxic reaction to the drugs. Fifth, lipophilic drugs penetrate the blood vessels in a manner similar to that of fat embolism and induce physical occlusion [8].

The typical presentation is blanching and pain around the infection site soon after injection, followed by erythema, livedoid patch, hemorrhagic patch and finally necrosis [2]. Secondary infection might occur [2].

Diagnosis is mainly clinical; a skin biopsy shows necrotic changes caused by ischemia [9].

Additional treatment includes antibiotics, wound dressing, skin graft, and flap reconstruction; extensive scarring is usually inevitable [9]. Early treatment has been reported to avert necrosis of the skin [10]. Surgical debridement of the ulcer is of the utmost importance as it reduces infection and enhances wound healing [10]. Systemic antibiotics play a vital role in the management of this syndrome. In our case, the secondary staphylococcal infection was treated effectively with antibiotics. According to Uri and Arad [11], various studies reported the clinical improvement of patients that had being treated with anticoagulants (such as heparin), intravenous steroids (such as betamethasone, dexamethasone or intravenous methylprednisolones) and vasoactive therapy (such as pentoxifylline) [11]. According to Murthy *et al.* conservative treatment with debridement and pain control is the main therapy [12]. Late complications include contractures and deformities resulting from the scarring process that require corrective surgery [12]. There are neurological complications, usually transitional, in one-third of patients, most commonly hypoesthesia and paraplegia [12]. Studies are needed to determine the pathogenesis mechanisms and recommended treatment procedures of NS. However, the possibility of forming a large series does not seem to be likely. To the best of our knowledge, our patient is the 30^th^ NS case associated with diclofenac sodium in the literature.

NS is an avoidable complication. Although the onset of NS cannot be predicted, in order to reduce the risk of tissue damage as much as possible, the injection is administered to the superolateral region of the gluteal muscle, for which an injection needle is used that is long enough to reach the muscle. The Z-track injection is a method of intramuscular injection into a large muscle using a needle and syringe and it can minimize or prevent NS [13]. Health care personnel should take these precautions. First, a long needle (long enough to reach the muscle) should be used. A 90-kg patient requires a 5 to 7.5cm (2- or 3-inch) needle and a 45-kg patient requires a 3.18 or 3.68cm (1.25- or 1.45-inch) needle. Second, the injection should be applied in the upper outer quadrant of the buttock. Third, aspirating the needle before injecting the medication should be performed to ensure that no blood vessel is hit. Fourth, the health care personnel should never inject more than 5ml of medication at a time when using the Z-track injection method. Finally, if more than one injection or a larger dose is required or ordered, different sites should be chosen [9, 13].

## Conclusions

Health care personnel should be aware that NS, characterized by pain, skin discoloration, and necrosis, can be observed following an injection of diclofenac sodium, an NSAID. As the exact etiopathogenesis of this syndrome is not known, there is no standard guideline for its management. Systemic antibiotics, wound debridement in early stages, and corrective plastic surgery in late stages are the mainstay in management. Clinicians should be aware of this complication and use proper injection procedures.

## Consent

Written informed consent was obtained from the patient for publication of this case report and any accompanying images. A copy of the written consent is available for review by the Editor-in-Chief of this journal.

## Abbreviations

HIV: Human immunodeficiency virus
NS: Nicolau syndrome
NSAID: Non-steroidal anti-inflammatory drug.
